# Association between oral health-related quality of life and physical frailty among community-dwelling older adults: A 2-year longitudinal study

**DOI:** 10.1016/j.tjfa.2024.100008

**Published:** 2025-01-01

**Authors:** Satoko Kakuta, Masanori Iwasaki, Yumi Kimura, Takatoshi Hiroshimaya, Ji-Woo Park, Taizo Wada, Yasuko Ishimoto, Michiko Fujisawa, Kiyohito Okumiya, Kozo Matsubayashi, Ryuji Hosokawa, Hiroshi Ogawa, Ryota Sakamoto, Toshihiro Ansai

**Affiliations:** a Division of Community Oral Health Development, Kyushu Dental University, Kitakyushu, Japan; b Division of Preventive Dentistry, Department of Oral Health Science, Graduate School of Dental Medicine, Hokkaido University, Sapporo, Japan; c Department of International Cooperation and Multicultural Studies, Tsuda University, Tokyo, Japan; d Department of Preventive Dentistry, Kagoshima University Graduate School of Medical and Dental Sciences, Kagoshima, Japan; e Center for Southeast Asian Studies, Kyoto University, Kyoto, Japan; f Department of Health and Sports Science, Faculty of Health Science and Technology, Kawasaki University of Medical Welfare, Kurashiki, Japan; g Wildlife Research Center, Kyoto University, Kyoto, Japan; h Division of Oral Reconstruction and Rehabilitation, Kyushu Dental University, Kitakyushu, Japan; i Division of Preventive Dentistry, Department of Oral Health Science, Niigata University Graduate School of Medical and Dental Sciences, Niigata, Japan

**Keywords:** Oral health-related quality of life, Frailty, Longitudinal studies, Older adults, Subjective oral health

## Abstract

**Background:**

Frailty is a major health concern among older adults, and its association with oral health-related quality of life (OHRQoL) remains underexplored in longitudinal studies.

**Objective:**

To investigate the association between baseline OHRQoL and physical frailty incidence at a 2-year follow-up in community-dwelling older adults.

**Design:**

Prospective longitudinal study.

**Setting:**

The study was conducted within the Tosa Longitudinal Aging Study framework in Japan.

**Participants:**

This study included 144 community-dwelling older adults (50 men and 94 women; median age, 81.0 years) with complete data who participated in the Tosa Longitudinal Aging Study in 2016 and 2018 and were not categorized as physical frailty in 2016.

**Measurements:**

Baseline assessment included OHRQoL, which was evaluated using the General Oral Health Assessment Index (GOHAI; range 12–60), with higher scores indicating better OHRQoL, oral function, and general health status. The incidence of physical frailty was defined using the revised Japanese version of the Cardiovascular Health Study criteria. The association between the GOHAI score and physical frailty was assessed using logistic regression analysis.

**Results:**

The median baseline GOHAI score was 58. The incidence of frailty after a 2-year follow-up was 13.9 % among the participants (18.0 and 11.7 % for men and women, respectively). Each point of the GOHAI score was associated with an 11 % reduction in frailty risk over 2 years after adjusting by age, sex, number of teeth, Food Diversity Score, Geriatric Depression Scale score, eating alone, smoking, and more than five medications (adjusted odds ratio: 0.893; 95 % confidence interval: 0.810–0.984).

**Conclusions:**

This longitudinal study showed that a higher baseline OHRQoL, based on the GOHAI score, was linked to a lower incidence of physical frailty among community-dwelling older adults after 2 years.

## 1 Introduction

Frailty is a state of increased vulnerability to impaired homeostasis following a stressor event, which increases the risk of adverse outcomes [[1], [2], [3]]. The rapidly increasing aging population worldwide has led to more older adults experiencing frailty. Frailty is considered a reversible condition in the intermediate stage between being healthy and needing long-term care; therefore, prevention, early detection, and intervention in older adults are important [1,3,4]. In aging societies, rising healthcare costs pose critical challenges, underscoring the need for early detection and prevention to help manage these costs [5].

Oral health is vital for maintaining general health in older adults [6,7]. Oral functions such as eating, swallowing, and speaking play an important role in daily living [[8], [9], [10]]. Decline in these functions can affect food choices, reduce appetite, and impair social interaction. In older adults, reduced oral function is strongly related to the onset and progression of frailty, which is a leading cause of mortality [[1], [2], [3], [4],^7^,^11^,^12^]. Furthermore, chronic undernutrition leads to sarcopenia, characterized by a loss of muscle strength and mass, which is associated with frailty [[13], [14], [15]].

Oral health can be evaluated in two ways: 1) objective evaluation, in which dentists measure, observe, and quantitatively evaluate participants during health checkups and surveys, and 2) subjective evaluation, in which the participants’ thoughts and feelings are evaluated using questionnaires. Compared to the objective measurement method, using questionnaires can reduce the cost of health checkups and surveys. The General Oral Health Assessment Index (GOHAI), developed by Atchinson et al., assesses oral health-related quality of life (OHRQoL), including physical and psychosocial functioning, pain, and discomfort [16]. Various countries have translated their versions, and the validity of the Japanese version has been verified [17].

Previous studies on oral function and its association with frailty have included various factors, such as the number of teeth and severe periodontal disease [18], functional dentition [13], bite force [10], edentulism [19], poor oral health [20], lower articulatory oral motor skill [21], and xerostomia [22]. These studies primarily used objective measures and linked these factors with frailty in longitudinal studies of community-dwelling older adults [10,13,18,[20], [21], [22], [23]].

Several studies have reported the association between frailty and a combination of subjective questions on oral status and objective oral function measures [7,12]. In addition, a few cross-sectional studies have examined the association between OHRQoL and frailty [11,24]. However, a clear association between GOHAI scores and future physical frailty has not been reported longitudinally.

Motoishi et al. demonstrated that the OHRQoL was associated with physical frailty using the GOHAI scores in a cross-sectional study [11]. However, no longitudinal studies have explored this association using the GOHAI. Therefore, this longitudinal study examined the association between GOHAI and frailty, aiming to determine how higher baseline GOHAI scores (indicating better OHRQoL) are associated with physical frailty.

## 2 Methods

### 2.1 Study design and participant population

**Fig. 1:**
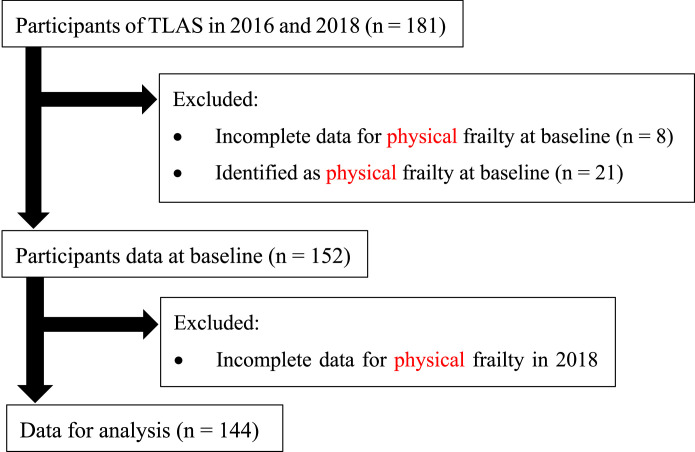
Flow chart of participant populations.

This prospective secondary analysis used data from the Tosa Longitudinal Aging Study (TLAS) [25], which surveyed the general health and physical functions, such as cognitive function and daily activities, among community-dwelling older people aged ≥65 years since 2004. Tosa town, located in Shikoku, southwestern Japan, has a population of approximately 3,500 and is primarily engaged in agriculture and forestry. The aging rate is over 45 %, and the town is involved in this original health checkup program (TLAS) as part of its medical, welfare, and long-term care services. Basically, all residents are covered by the public health care system. In TLAS, all community-dwellers aged ≥75 years are invited to participate. Dental-related measurements were introduced to TLAS in 2010. This study included 181 community-dwelling older adults who participated in the TLAS in 2016 and 2018, using 2016 as the baseline. Adults who were frail at baseline (21 participants) or had missing baseline data on frailty in 2016 and 2018 (8 participants each) were excluded, and the occurrence of frailty as of 2018 was examined. We analyzed 144 participants (Fig. 1).

This study was conducted according to the guidelines of the Declaration of Helsinki. All procedures involving human participants were approved by the Ethics Committee of the Faculty of Medicine, Kyoto University, Japan (approval number: C1292). Written informed consent was obtained from all the participants.

### 2.2 General (Geriatric) oral health assessment index (GOHAI)

The GOHAI, comprising 12 questions, has been validated for reliability in older populations [17]. It comprises three dimensions: physical functioning (five questions), psychosocial functioning (five questions), and pain and discomfort (two questions). Each question was answered on a 5-point Likert scale from “always” (1 point) to “never” (5 points). The Japanese version features only negatively worded questions [17]. The cumulative score (range 12–60 points) reflects OHRQoL, with higher scores indicating a better quality.

### 2.3 Physical frailty assessment

Frailty assessment was based on the revised Japanese version of the Cardiovascular Health Study (J-CHS) criteria, which include five components: shrinking, weakness, exhaustion, slowness, and low activity [26]. Weight loss for shrinking was defined as an affirmative response to the question, “In the last 6 months, have you lost 2–3 kg or more?” Grip strength for weakness was measured once for each hand using a handheld dynamometer (T.K.K. 5401, Takei Scientific Instruments, Niigata, Japan), with a higher value used for analysis [27]. The cut-off values for grip strength were <28 kg and <18 kg for men and women, respectively. Exhaustion was defined as an affirmative response to the question, “feel tired for no reason (in the past 2 weeks)?” Gait speed for slowness was measured using a 4-m walking test. Participants walked over a 4-m course on a flat floor at their usual speed, and the time required for walking was recorded with a stopwatch. Gait speed (m/s) was calculated by dividing distance (m) by time (s) [27]. The cut-off value for slowness was <1.0 m/s. Low activity was determined by a negative response to both “Do you engage in light physical exercise or sports?” and “Do you engage in regular exercise?” Physical frailty was defined as meeting three or more of these criteria using data from 2016 to 2018.

### 2.4 Oral and dietary assessments

At the baseline survey, oral function was assessed through several measures. Dental status, including the number of teeth and denture use (upper or lower dentures), were recorded by trained dentists. Masticatory function was assessed using a color-changing gum (XYLITOL, 3.0 g; Lotte, Saitama, Japan) and the CR-13 Color Reader (Konica Minolta Holdings, Inc., Tokyo, Japan). Participants were asked to chew for 1 min at their usual speed. The change in gum color (colorimeter ΔE*ab) before and after chewing was calculated as described previously [27,28], with lower ΔE*ab indicating poor masticatory function. Swallowing function was assessed using the repetitive saliva-swallowing test (RSST) [29], where participants were asked to perform repetitive voluntary swallowing as quickly as possible for 30 s. Swallows were counted and used as the RSST scores, and three or more swallows in 30 s were considered normal. Oral diadochokinesis (Pa, Ta, and Ka), an index of tongue and lip motor dexterity, was assessed using the Kenko-kun® (T.K.K. 3350 digital counter, Takei Scientific Instruments, Niigata). Each sound was repeated as quickly as possible for 5 s, and the total number of times counted using a digital counter in 5 s was expressed in times per second [30]. When each sound was < 6 times/s, the participants were considered to have a dysfunctional tongue and lip movement.

The Simplified Nutritional Appetite Questionnaire (SNAQ) is a four-item appetite questionnaire, with a score ≤14 defined as poor appetite [31]. In addition, dietary habits were evaluated using the 11-item Food Diversity Score Kyoto (FDSK-11) [32]. FDSK-11 comprises 11 main food groups (grain, meat, fish and shellfish, eggs, milk, beans and soybean products, potatoes, vegetables, seaweed, nuts, and fruits). Participants reported if they had eaten each of the 11 food groups once or more in a week (score = 1) or less than once in a week (score = 0). The summed score indicates a FDSK-11 score (range: 0–11, with higher scores indicating greater food diversity), and scores were classified into two groups based on a 9–10 cutoff as in a previous study [32].

### 2.5 General conditions and lifestyle assessments

Body composition was measured using the Inbody S10 (Inbody Japan, Tokyo), a bioelectrical impedance analysis method, in which the skeletal muscle index (kg/m^2^) was calculated by dividing the weight of the limb skeletal muscle (kg) by the square of height (m^2^) [27]. Body mass index (BMI) was calculated (kg/m^2^), with underweight defined as BMI ≤18.5 kg/m^2^. Disease-related items such as more than five medications, medical history, depressive symptoms, and cognitive impairment status were assessed using the self-rated questionnaires. A number of medications were addressed in an affirmative response to the question, “Are you taking more than five medications?” For medical history, participants answered about the presence of specific diseases and current hospital visits. Depressive symptoms were measured using the five-item Geriatric Depression Scale (GDS-5 range: 0–5; higher scores indicate severe depression), with scores ≥2 indicating depression. Cognitive status was assessed by the trained examiner using the Mini-Mental State Examination (MMSE; range: 0–30, with lower scores indicating a decline in cognitive function). Cognitive impairment was defined as an MMSE score ≤23. Lifestyle habits such as smoking and alcohol consumption were assessed using a self-rated questionnaire, with past and current smokers categorized as smokers. For alcohol consumption, those who consumed alcohol occasionally or daily were considered to have drinking habits.

### 2.6 Statistical analysis

The outcome variable was the 2 year incidence of frailty based on J-CHS criteria. The association between baseline GOHAI score and frailty incidence was analyzed using univariate and multivariate logistic regression models. The multivariate model included covariates determined based on previous studies [2,11] and a univariate model. In this study, three models were constructed: Model 1 included the GOHAI scores, age, and sex; Model 2 was adjusted for the variables relevant in univariate analysis (*p* < 0.2); Model 3 was fully adjusted for potential confounders. Collinearity was minimized by excluding the two correlated variables. All analyses were performed using SPSS (ver. 28) (IBM Japan, Tokyo), and statistical significance was defined as *p* < 0.05.

## 3 Results

Table 1 presents the baseline characteristics of the study population and the results of the crude association between these characteristics and physical frailty incidence after 2 years. The participants had median age of 81 years, with 65.3 % women and an overall 13.9 % 2-year incidence of frailty. The median GOHAI scores were 58 and 56.5 in the non-frail and frail groups, respectively. Participants in the frail group were generally older and more likely to be male compared to the non-frail group. In terms of oral health and nutrition, they had lower GOHAI scores (lower OHRQoL, in all dimensions), less appetite, fewer teeth, lower masticatory function, a higher percentage of denture use, lower tongue dexterity for all “pa,” “ta,” and “ka” sounds, and less food diversity. Furthermore, in terms of lifestyle, the participants were less likely to live or eat alone and had a history of smoking. General conditions included depression, cognitive decline, decreased skeletal muscle mass, and use of multiple medications.

**Table 1:** Crude association of baseline characteristics with physical frailty incidence.

	Overall *n* = 144	Non-frail *n* = 124 (86.1)	Frail *n* = 20 (13.9)	OR_crude_ (95 % CI)	p*
Age (years), median (IQR)	81 (79–85)	81 (78–84)	84 (81–87.75)	1.137 (1.031–1.255)	**0.010**
Gender, (%)					
Men	34.7	33.1	45.0	1	
Women	65.3	66.9	55.0	0.604 (0.232–1.572)	0.301
**Subjective measurements**					
GOHAI, median (IQR)	58 (54–60)	58 (55–60)	56.5 (48–58)	0.888 (0.8200–962)	**0.004**
GOHAI dimensions, median (IQR)					
Physical functioning	24 (22–25)	25 (23–25)	23 (17.3–25)	0.805 (0.703–0.923)	0.002
Psychosocial functioning	25 (23–25)	25 (23–25)	24 (21.3–25)	0.849 (0.723–0.996)	0.045
Pain and disconfort	10 (10–10)	10 (10–10)	10 (8.5–10)	0.675 (0.435–1.046)	0.079
SNAQ score, median (IQR)	15 (14–16)	15 (14–16)	15(14–16)	0.873 (0.620–1.229)	0.436
Poor appetite ≤14, (%)	29.9	29.0	35.0	1.316 (0.486–3.568)	0.589
**Objective measurements**					
Number of teeth, median (IQR)	16 (5–24)	17 (6–24)	9 (0–22)	0.968 (0.923–1.014)	0.170
Number of teeth ≤19, (%)	41.3	43.1	30.0	1.767 (0.637–4.903)	0.275
Number of teeth ≥ 20	58.7	56.9	70.0	1	
Masticatory function (ΔE*ab) , median (IQR)	53.6 (47.7–57.8)	53.8 (48.9–58.0)	50.7 (41.8–56.2)	0.951 (0.912–0.991)	**0.017**
RSST ≤2, (%)	20.3	20.2	20.0	1.057 (0.980–1.139)	0.150
Denture use, (%) (n = 143)	65.0	62.9	75.0	1.731 (0.590–5.079)	0.308
Oral diadochokinesis (times/second)					
“Pa” sound	5.6 (4.8–6.4)	5.6 (5.0–6.4)	5.1 (3.9–6.4)	0.745 (0.513–1.083)	0.123
“Ta” sound	5.4 (4.8–6.4)	5.6 (4.8–6.4)	5.1 (4.8–5.9)	0.721 (0.492–1.055)	0.092
“Ka” sound	5.2 (4.4–5.8)	5.2 (4.6–5.8)	4.4 (3.9–5.4)	0.585 (0.384–0.893)	**0.013**
FDSK-11,median (IQR)	11 (10–11)	11 (10–11)	10 (9–11)	0.735 (0.501–1.078)	0.085
FDSK-11 ≤9, (%)	22.9	19.4	45.0	3.409 (1.270–9.149)	**0.015**
FDSK-11 ≥10	77.1	80.6	55.0	1	
Depressive symptoms, (%) (n = 135)					
GDS-5 ≥2	23.0	20.2	35.0	2.147 (0.7656.023)	0.147
GDS-5 <2	77.0	79.8	65.0	1	
Cognitive impairment, (%)					
MMSE ≤23	88.1	90.3	80.0	2.146 (0.616–7.476)	0.231
MMSE ≤23	11.9	9.7	20.0	1	
Lifestyle, ( %)					
Living alone (yes)	27.8	29.8	15.0	0.432 (0.118–1.586)	0.206
Eating alone (yes)	37.5	39.5	25.0	0.488 (0.165–1.446)	0.196
Smoking status (previous, current)	25.0	23.4	35.0	1.764 (0.643–4.836)	0.270
Drinking habit (sometimes, everyday)	46.5	46.0	50.0	1.175 (0.457–3.024)	0.737
General conditions, ( %)					
SMI (kg/m^2^) , median (IQR)	6.2 (5.6–7.1)	6.2 (5.6–7.1)	6.3 (5.5–7.2)		
Men (n = 49)	7.2 (6.7–7.7)	7.3 (6.8–7.7)	7.2 (6.3–7.8)		
Momen (n = 92)	5.9 (5.5–6.3)	5.9 (5.5–6.3)	5.8 (5.0–6.3)		
BMI <18.5kg/m^2^ (yes)	4.2	4.0	5.0	1.253 (0.139–11.316)	0.841
More than 5 kinds of medications (yes)	27.1	25.0	40.0	1.892 (0.708–5.062)	0.204
Diabetes Mellitus (yes)	7.6	7.3	10.0	1.420 (0.284–7.108)	0.670
Heart disease (yes)	11.1	12.1	5.0	0.382 (0.048–3.068)	0.366
Cerebrovascular disease (yes)	4.2	4.0	5.0	1.253 (0.139–11.316)	0.841
Articular disease (yes)	36.8	37.1	35.0	0.913 (0.340–2.453)	0.857

IQR, interquartile range; GOHAI, general oral health assessment index; SNAQ, Simplified Nutritional Appetite Questionnaire; RSST, repetitive saliva swallowing test; FDSK-11, food diversity score Kyoto-11; GDS-5, geriatric depression scale-5; MMSE, mini-mental state examination; SMI, skeletal muscle index; BMI, body mass index; Oral data n = 143. *p-value for the 95 % CI of odds.

The result of the crude association between baseline characteristics and physical frailty showed that each 1-year increase in age is associated with a 1.1-fold risk of frailty over 2 years. Each additional GOHAI point was associated with an 11 % reduction in frailty risk, and for the GOHAI dimension, each one-point increase in the physical and psychosocial dimensions was associated with 19 and 15 % lower risks, respectively. Higher masticatory ability and faster repetition of "Ka" sounds were associated with 5 and 41 % lower risks, respectively. Furthermore, participants with low dietary diversity (FDSK-11 ≤9) were 3.4 times more likely to develop frailty than those with high dietary diversity (FDSK-11 ≥10).

Table 2 presents the logistic regression analysis for physical frailty incidence after 2 years. The final model (Model 3) adequately described the data (Hosmer‒Lemeshow goodness-of-fit test: 0.114), explained 27.4 % of the frailty variability (Nagelkerke pseudo r-squared), and 84.9 % of the participants were classified properly. Each additional GOHAI point was associated with a 12 % lower incidence of physical frailty over 2 years in the crude analysis, and this association remained at 11 % after adjusting for all relevant variables included in the study. Notably, participants with lower dietary diversity had a 4.8 times higher incidence of physical frailty over 2 years compared to those with greater dietary variety.

**Table 2:** Adjusted associations of GOHAI with physical frailty incidence

	Model 1			Model 2			Model 3		
Variables	ORs	95 % CIs	*p*	ORs	95 % CIs	*p*	ORs	95 % CIs	*p*
GOHAI (per one point increase)	0.892	0.821–0.970	0.007	0.894	0.812–0.985	0.023	0.893	0.810–0.984	0.023
Age (per one increase) Women (versus men)	1.119 0.533	1.010–1.240 0.189–1.505	0.031 0.235	1.120 0.561	0.993–1.264 0.182–1.722	0.065 0.312	1.132 0.634	0.998–1.282 0.143–2.805	0.053 0.548
Number of teeth (median) FDSK-11 ≤9 GDS-5 ≥2 Eating alone (yes)				1.012 4.364 1.081 0.390	0.951–1.073 1.342–14.197 0.302–3.872 0.112–1.351	0.688 0.014 0.904 0.137	1.017 4.847 1.008 0.368	0.957–1.081 1.403–16.746 0.269–3.784 0.104–1.308	0.588 0.013 0.990 0.122
Smoking status							1.061	0.222–5.065	0.941
More than 5 kinds of medications (yes)							1.044	0.301–3.626	0.946

OR, odds ratio; CI, confidence intervals; GOHAI, general oral health assessment index; FDSK-11, food diversity score Kyoto-11; GDS, geriatric depression scale.

## 4 Discussion

This study showed that each additional point in the GOHAI score was associated with an 11 % lower incidence of frailty over 2 years among community-dwelling older adults using a public service health screening scheme in rural Japan, even after adjusting for relevant variables. The GOHAI score includes physical functioning, psychosocial functioning, pain, and discomfort as subscales for assessing OHRQoL. In the GOHAI dimension, physical functioning and psychosocial dimensions were shown to have crude associations with physical frailty. In this study of 144 community-dwelling older adults, participants with higher baseline GOHAI scores, indicating better OHRQoL, had a lower incidence of frailty 2 years later (OR: 0.893) compared to participants with lower GOHAI scores, even after adjusting for potential covariates. To our knowledge, this is the first longitudinal study to demonstrate an association between physical frailty and OHRQoL using the GOHAI score.

Several studies have reported an association between subjectively assessed oral status and frailty in both cross-sectional and longitudinal analyses. Motoishi et al. [11] reported a cross-sectional association between the GOHAI score as a measure of OHRQoL and physical frailty among 72 year-old community-dwelling Japanese. Ramsey et al. [12] and Tanaka et al. [7] demonstrated the association between oral status and frailty using self-rated questionnaires on “difficulty eating” and “dry mouth symptoms.” Our findings align with these studies [7,11,12], indicating that the oral health status assessed through subjective measurements is associated with physical frailty.

One possible mechanism for the association between OHRQoL and physical frailty is a pathway through oral functional status, nutritional intake, and the immune system. The frailty cycle proposed by Fried et al. states that chronic malnutrition mainly influences the onset of frailty [4,11]. Previous Japanese studies have shown that low masticatory ability is associated with low serum albumin levels (a marker of malnutrition) [33] and that low protein intake is associated with frailty development [34]. Poor oral health, even if self-perceived, may increase markers of inflammation, affecting the metabolism of other organs and increasing frailty risk [6].

The second possible mechanism by which OHRQoL influences physical frailty involves nutritional factors and social activities. Reduced OHRQoL may lead to anxiety about appearance and reluctance to engage in social activities owing to diet-related problems, thereby increasing frailty risk [35,36]. Malnutrition and poor nutritional status are also associated with the onset and progression of frailty [37,38]. The psychosocial dimension of the GOHAI includes questions about social interactions and eating in front of people. Those with a low score on these questions may have poor social contact and a high risk of physical frailty [11,39]. Thus, these findings suggest that frailty is not only associated with objective indices of poor oral function and nutrition, as previously reported [2,10,21] but also with subjective OHRQoL as assessed by GOHAI, which represents oral abnormalities and discomfort.

Another possible mechanism for this association between OHRQoL and frailty is the number of denture wearers satisfied with stable dentures. In this study, 65.0 % of the participants wore dentures (upper full dentures, 33.6 %; lower full dentures, 22.4 %). Previous studies have shown positive associations between OHRQoL and denture satisfaction among denture wearers [[40], [41], [42]]. These results suggest that masticatory ability and oral status (whether with partial or complete dentures) determine denture satisfaction and are mostly associated with the OHRQoL. Yamaga et al. [42] concluded that patients with more stable mandibular complete dentures can eat easily and have fewer oral health problems, contributing to a high OHRQoL. Moreover, the high risk of frailty in edentulous individuals without dentures may be owing to dietary changes (such as subtle changes in dietary choices, reduced nutrient intake, and increased saturated fat intake) that alter the physiological response of an organism to internal and external stressors, leading to weakness and slowness [19]. Further detailed information on dentures is needed to understand the association between dentures and frailty incidence.

Applying these mechanisms to this study, the study population had few teeth but maintained good oral function, as indicated by a stable denture fit and high dietary diversity (FDSK-11), allowing them to eat various foods. Furthermore, their diet may have contributed to good OHRQoL, stable muscle mass, and physical activity such as farm work (Tosa town is a farming village surrounded by mountains), leading to more opportunities for social engagement and less fatigue.

The strength of this longitudinal study is that it examined the relationship between OHRQoL, using the GOHAI score, and the development of physical frailty. While previous studies examining the association between GOHAI scores and frailty were cross-sectional, this study demonstrates that subjective oral status, as assessed by the GOHAI score, is associated with the development of physical frailty after 2 years.

However, this study had some limitations. First, it was conducted in a rural area of Japan among older adults aged ≥75 years, limiting the generalizability of the results. Second, the target population and sample size were small (target population: n = 925). Sampling was conducted through a municipal public service health screening scheme. All residents aged ≥75 years were invited; however, only those who voluntarily attended were included. In previous years, only approximately 30 % of eligible participants attended (267 participants; 28.8 % in 2016). Although the final model (Model 3) was statistically meaningful, the power was inferior to previous studies that examined oral health and frailty [6,19], probably due to the small sample size. Further large-scale surveys are required to confirm these results. Third, the study lacked data on social and environmental factors, such as household composition and educational background, preventing adjustment for these variables. A recent review identified that determinants, such as low educational level and marital status, are associated with poor OHRQoL [43]. Their study showed that tooth replacement may have an economic impact. Therefore, various background factors should be considered when assessing OHRQoL associations. Fourth, because this study used the Fried et al. phenotypic model as its definition of frailty, it primarily assessed physical aspects in determining frailty and did not account for cognitive and other psychosocial factors.

This study used the GOHAI to assess OHRQoL. The GOHAI is a non-invasive, easily assessed frailty screening tool suitable for clinical and community settings [11], requiring only a few questions and no equipment. According to Castrejón-Pérez et al. [44], oral health problems accumulate throughout life, and even if they are initially low-intensity and ignorable, they may progress until very complicated if not addressed at the appropriate time. They noted that the utilization of dental services and self-perception of oral health are associated with a higher probability of frailty [6]. In summary, early recognition of subjective oral problems (including pain, discomfort, or appearance) in older adults during community health examinations and clinical practice, followed by early intervention by professionals and municipal officials, may help to prevent or control nutritional and systemic problems that can lead to frailty and subsequent adverse outcomes. Educating older adults to address minor oral health problems promptly is also important.

## 5 Conclusion

This study examined, longitudinally, the association between OHRQoL using the GOHAI score and the incidence of physical frailty among community-dwelling older adults. The results showed that higher baseline GOHAI scores (indicating better OHRQoL) were significantly associated with a lower incidence of physical frailty at 2 years.

## Informed consent statement

Written informed consent was obtained from all study participants.

## Funding

This work was partially supported by JSPS KAKENHI (Grant Numbers 16KT0120, 16K11868, 19K10449, and 21H03134).

## CRediT authorship contribution statement

Satoko Kakuta: Conceptualization, formal analysis, investigation, writing – original draft, writing – review and editing. Masanori Iwasaki: Data curation, formal analysis, investigation, writing, reviewing, and editing. Yumi Kimura: Data curation, investigation, writing, review, and editing. Takatoshi Hiroshimaya: Investigation. Ji-Woo Park: Investigation. Taizo Wada: Investigation, writing, review, and editing. Yasuko Ishimoto: Investigation and resources. Michiko Fujisawa: Investigation and resources. Kiyohito Okumiya: Investigation and supervision. Kozo Matsubayashi: Investigation and supervision. Ryuji Hosokawa: Investigation and resources. Hiroshi Ogawa: Investigation and resources. Ryota Sakamoto: Investigation and project administration. Toshihiro Ansai: Conceptualization writing, review, and editing.

## Data management and sharing

This research lacks supporting data owing to its nature.

## Declaration of competing interest

The authors declare no conflict of interest.
